# Two Uncomplicated Pregnancies on Alectinib in a Woman With Metastatic *ALK*-Rearranged NSCLC: A Case Report

**DOI:** 10.1016/j.jtocrr.2022.100361

**Published:** 2022-06-18

**Authors:** Chloe Weidenbaum, Christopher G. Cann, Sarah Osmundson, Wade T. Iams, Travis Osterman

**Affiliations:** a Department of Medicine, University of Tennessee Health Science Center, Nashville, Tennessee; b Division of Hematology-Oncology, Department of Medicine, Vanderbilt University Medical Center, Nashville, Tennessee; c Department of Obstetrics and Gynecology, Vanderbilt University Medical Center, Nashville, Tennessee; d Vanderbilt-Ingram Cancer Center, Nashville, Tennessee

**Keywords:** *ALK-*rearranged non–small cell lung cancer, Pregnancy, Case report, Alectinib

## Abstract

Lung cancer incidence is increasing in pregnancy due in part to advanced maternal age. A subset of patients with NSCLC during pregnancy harbor an *ALK* gene rearrangement. Although ALK inhibitors, such as alectinib, are routinely used to treat *ALK*-rearranged NSCLC, there are limited safety data regarding use during pregnancy and fetal effects. Here, we report the second case of a patient with metastatic *ALK*-rearranged lung adenocarcinoma treated with alectinib throughout pregnancy. Notably, the patient had two uncomplicated pregnancies with routine obstetrical and postnatal courses. In this case, alectinib did not seem to affect embryofetal or early childhood development. This does not exclude undetectable or delayed toxic effects, and additional studies are needed to further reveal the safety of alectinib treatment during pregnancy.

## Introduction

The incidence of lung cancer during pregnancy is increasing partly due to advancing maternal age.^1^ A subset of NSCLC exhibit *ALK* gene rearrangements. There is an association between the presence of an *ALK* rearrangement and NSCLC during pregnancy, likely due in large part to the higher incidence of *ALK* rearrangements in women of childbearing age.^2^

The *ALK* gene encodes a transmembrane tyrosine kinase receptor expressed during embryogenesis and thought to play a role in nervous system development. Although ALK inhibitors are routinely used to treat *ALK*-rearranged NSCLC, there are limited safety data regarding use during pregnancy and fetal effects.^2^^,^^3^ Here, we report the second case of a patient with metastatic *ALK*-rearranged lung adenocarcinoma treated with alectinib throughout pregnancy. This patient provided informed consent by means of Vanderbilt Institutional Review Board protocol #030763 for this case report.

## Case Presentation

**Figure 1:**
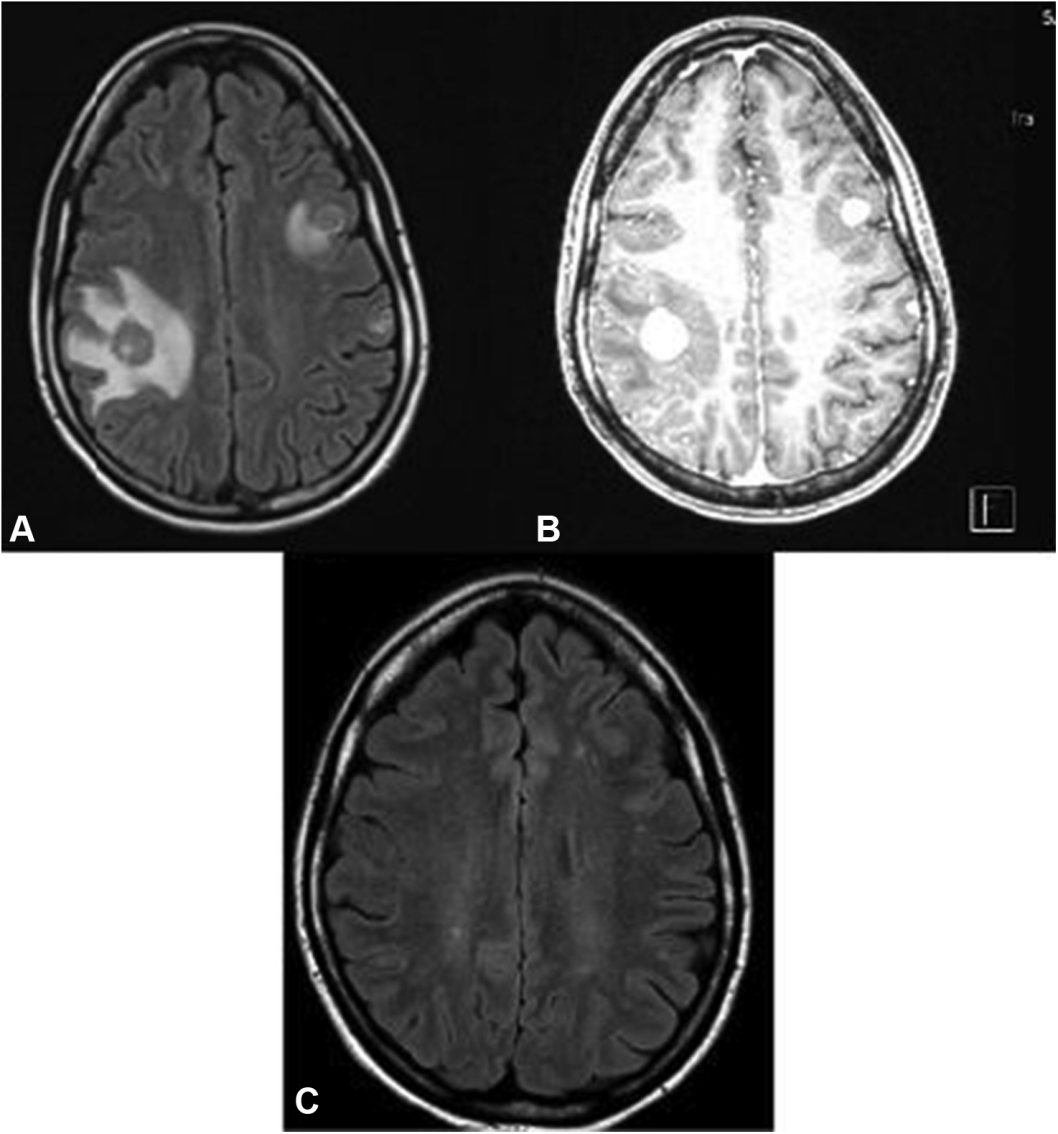
(*A*) MRI brain T2 flair at the time of diagnosis. (*B*) MRI brain with contrast at the time of diagnosis. (*C*) MRI brain with contrast 4.5 years after diagnosis. MRI, magnetic resonance imaging.

**Figure 2:**
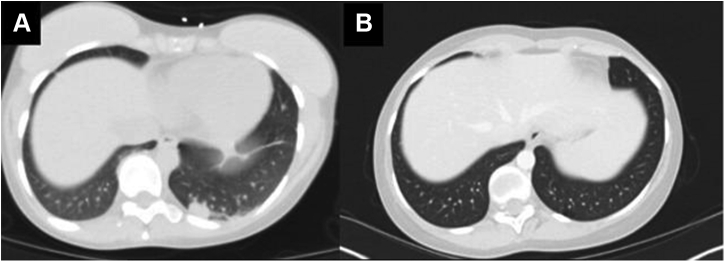
(*A*) CT chest with contrast at the time of diagnosis. (*B*) CT chest with contrast 4.5 years after diagnosis. CT, computed tomography.

A 27-year-old woman presented after a tonic-clonic seizure. Result of brain magnetic resonance imaging revealed multiple ring-enhancing lesions, and results of computed tomography of the chest and abdomen noted a 2 cm by 1.1 cm left lower lobe lung mass and mediastinal lymphadenopathy (Figs. 1*A–C* and 2*A* and *B*). Result of computed tomography-guided biopsy of a mediastinal lymph node with fluorescence in situ hybridization testing confirmed *ALK*-rearranged adenocarcinoma, with programmed death-ligand 1 at 35%, and no additional actionable mutations on OnkoSight next-generation sequencing assessment. She underwent stereotactic radiosurgery to the brain metastases, and alectinib 300 mg twice daily was initiated.

After 16 months, while on treatment with alectinib 300 mg twice daily with excellent disease control, the patient unexpectedly became pregnant. In conjunction with Maternal-Fetal Medicine (MFM) experts, the potential risks and benefits of proceeding with pregnancy and continuing alectinib were reviewed. This discussion included information on how first trimester exposure to antineoplastic agents may result in congenital malformations and spontaneous abortions. Also discussed were the limited data regarding alectinib effects on fetal development, noting that in animal studies fetal developmental abnormalities and miscarriage occurred at doses 2.7× to 4.5× those used in humans. A collaborative decision was made to continue with pregnancy and alectinib 300 mg twice daily. Her MFM doctors recommended early detailed ultrasound at 17 weeks and fetal echocardiogram at 22 weeks. The patient was followed with serial growth ultrasounds every four weeks, antenatal testing at 32 weeks, and close monitoring for anemia and thrombocytopenia.

The patient’s pregnancy was uncomplicated other than iron-deficiency anemia requiring intravenous iron supplementation. No fetal anomalies or growth restriction were identified. The patient continued on alectinib 300 mg twice daily without disease progression or adverse effects. Scheduled induction of labor at 39 weeks of gestation resulted in an uncomplicated vaginal birth of a male infant with Apgar scores of 8 and 9. The newborn’s postnatal course was complicated by hyperbilirubinemia, which self-resolved. The patient and newborn were discharged home within 48 hours of delivery.

Given her successful pregnancy while on alectinib and in conjunction with MFM colleagues, the patient planned and conceived a second pregnancy. A multidisciplinary decision was made to continue with standard maternal-fetal monitoring without additional imaging unless the patient developed symptoms concerning for fetal distress or demise and continuation of alectinib 300 mg twice daily. Pregnancy was again uncomplicated, and the patient had a successful spontaneous vaginal delivery of a healthy female infant at 39 weeks and 3 days, with initial Apgar scores of 9 and 9. The newborn’s postnatal course was uncomplicated, and the patient and newborn were discharged home within 48 hours of delivery. The patient elected not to breastfeed beyond the first few days postpartum given unknown levels of alectinib in breast milk.

## Placental Findings

The histopathological characteristics of the placentas of both children were evaluated after spontaneous vaginal delivery. No pertinent morphologic or pathologic changes were noted other than fetal membrane grade 1 acute chorioamnionitis in the first pregnancy. There was no evidence of carcinoma in either placenta.

## Child Development and Maternal Follow-Up

**Figure 3:**
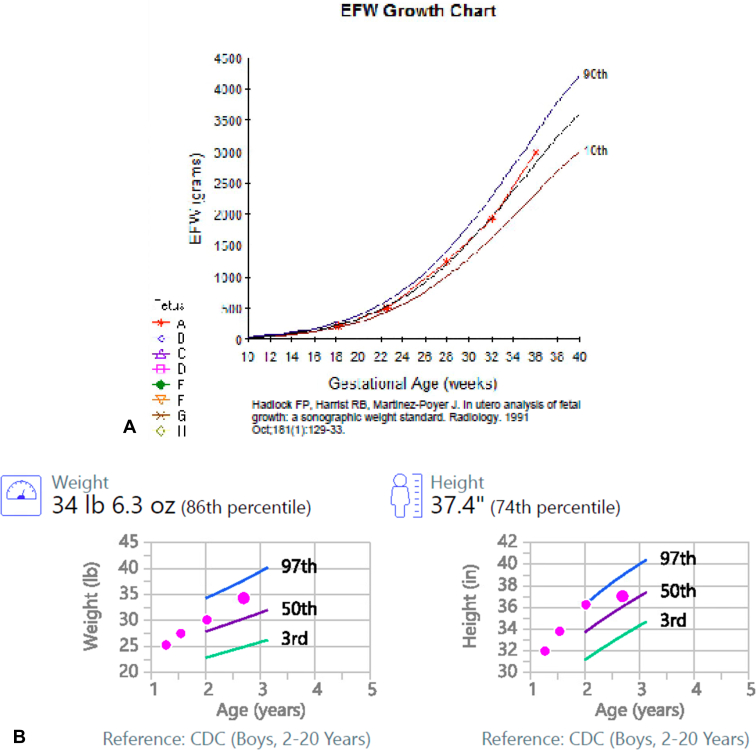

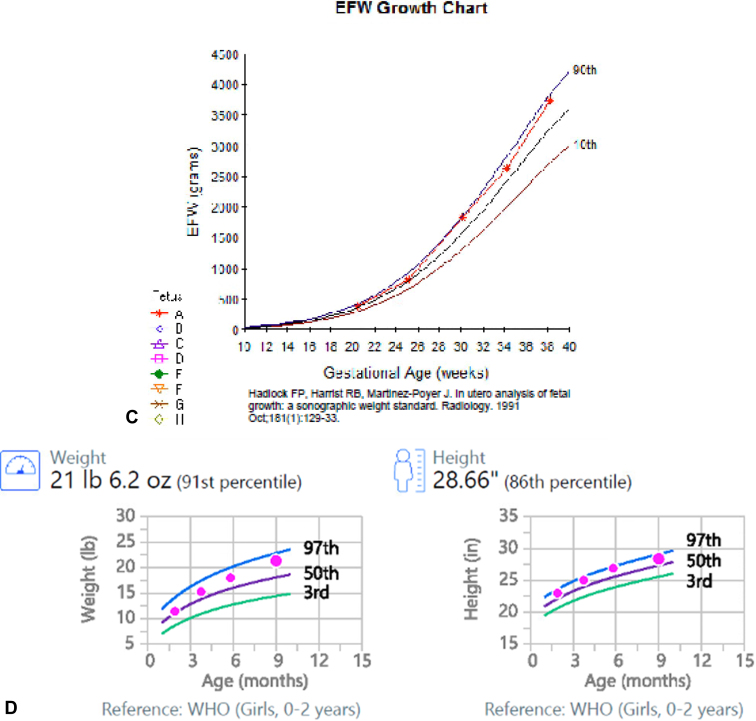
(*A*) Fetal growth curve for the first pregnancy. (*B*) Pediatric growth curves for the first pregnancy. (*C*) Fetal growth curve for the second pregnancy. (*D*) Pediatric growth curves for the second pregnancy. CDC, Centers for Disease Control and Prevention.

Both children experienced normal early childhood growth without indication for additional laboratory evaluation or imaging after delivery other than the standard of care. No congenital abnormalities were identified. At 2 years and 9 months and 10 months of age, both children exhibit normal growth and development for their respective ages without any underlying health conditions (Fig. 3*A–D*).

The patient has been on alectinib for more than 4 years with isolated progression of disease in the brain on one occasion, approximately three years after therapy initiation, which occurred three months after delivery of her second child. At the time of central nervous system progression, alectinib was increased from 300 mg twice daily to 600 mg twice daily, and the patient underwent additional stereotactic radiosurgery with an objective response to treatment (Fig. 1).

## Discussion

Pregnancy in patients with lung cancer is rare, although with treatment, young patients with *ALK*-rearranged NSCLC were found to have prolonged survival with good quality of life. Unfortunately, safety data regarding the teratogenicity of ALK inhibitors are lacking.^2^ There are currently only three reports of ALK inhibitor use during pregnancy, one with alectinib and two with crizotinib.^3^, ^4^, ^5^ Owing to the unique and complex nature of this clinical scenario, a multidisciplinary team was involved, including MFM and medical oncology. Cancer during pregnancy presents both a medical and ethical challenge, as treatment decisions affect two patients. For these reasons, a shared decision-making approach was emphasized.

In this case, alectinib was continued throughout the entirety of both pregnancies. There were no observed obstetrical complications. No postnatal complications were observed aside from self-limited newborn hyperbilirubinemia after the first pregnancy. Although in this case placental drug concentration was not measured, a previously reported case of alectinib use during pregnancy revealed low placental drug penetration and unaffected embryofetal development.^4^ In this case, no pertinent morphologic or pathologic placental changes were noted other than fetal membrane grade 1 acute chorioamnionitis in the first pregnancy, which is a common complication of labor. There was no evidence that alectinib affected embryofetal or early childhood development. Central nervous system imaging and a thorough neurocognitive evaluation for the children were unavailable. This, however, does not exclude undetectable or delayed toxic effects. Because of this, both children will continue to be monitored for the next several years for any developmental delays. Of note, the patient was initially started on half the maximum dose of alectinib to ensure tolerability. It is unclear at this time whether there could be a dose-dependent embryofetal effect.

## Conclusions

This case reveals that among a limited number of cases reported in the literature, alectinib treatment during pregnancy has not been associated with a negative impact on obstetrical course or embryofetal or early childhood development. Longer-term follow-up will be important to monitor for any late developmental effects, and additional studies are needed to further reveal the safety of alectinib treatment during pregnancy and breastfeeding. Fertile patients on ALK inhibitors should be informed of the existing drug safety data. It remains critical to manage cases like this with a multidisciplinary approach involving patient shared decision-making.

## CRediT Authorship Contribution Statement

**Chloe Weidenbaum:** Writing—original draft, Writing—review and editing, Visualization, Project administration.

**Christopher G. Cann:** Data Curation, Writing—original draft, Writing—review and editing.

**Sarah Osmundson:** Writing—review and editing.

**Wade T. Iams:** Conceptualization, Writing—review and editing, Supervision.

**Travis Osterman:** Writing—review and editing, Supervision.

## References

[1] Peccatori F.A., Azim H.A., Orecchia R. (2013). Cancer, pregnancy and fertility: ESMO Clinical Practice Guidelines for diagnosis, treatment and follow-up. Ann Oncol.

[2] Dagogo-Jack I., Gainor J.F., Porter R.L. (2016). Clinicopathologic features of NSCLC diagnosed during pregnancy or the peripartum period in the era of molecular genotyping. J Thorac Oncol.

[3] Scarfone G., Fumagalli M., Imbimbo M. (2021). First case report of pregnancy on alectinib in a woman with metastatic ALK-rearranged lung cancer: a case report. J Thorac Oncol.

[4] Padrão E., Melo C., Fernandes G. (2018). Lung cancer in pregnancy – report of a case treated with crizotinib. Pulmonology.

[5] Jensen K.H., Persson G., Storgaard L. (2019). Antineoplastic treatment with crizotinib during pregnancy: a case report. Acta Oncol.

